# 

**Published:** 1910-05-15

**Authors:** 


### ANNOUNCEMENTS.

NATIONAL ASSOCIATION OF DENTAL FACULTIES. The National Association of Dental Faculties will meet at the Hotel Shirley, Denver, Colo., Monday, July 25, at 10 o'clock A. M. The Executive Committee will meet to consider business of the association at 9 o'clock A. M., on the same day.

The Hotel Shirley is connected by bridge with the Savoy, where the meeting of the National Association of Dental Examiners takes place on the same date.

The rates will be $2.00 per day for one and $3.00 for two persons in one room, European plan. Large rooms for one or two persons with private bath, $4.00 and $5.00 per day.

B. HOLLY SMITH, *Chm'n Ex. Com.* N. A. D. F.,
Baltimore, Md.

INDIANA STATE DENTAL ASSOCIATION. The fifty-second annual meeting of the Indiana State Dental Association will be held in Indianapolis, May 17 to 19, 1910, at the Claypool Hotel. This promises to be a great meeting.

OTTO U. KING, *Sec'y*,

Huntington, Ind.

---

NATIONAL ASSOCIATION OF DENTAL EXAMINERS. Change of date of meeting. The twenty-eight anunal session of the National Association of Dental Examiners will be held at the new Savoy Hotel, Denver, Colo., commencing Monday, July, 25, 1910, at 10 A. M. The rates will be $2.00 per day for one and $3.00 per day for two persons in room, European plan; large room for one or two with private bath $4.00 and $5.00 per day. Meeting and committee rooms at the service of the association, free, and every accommodation extended.

An early mail reservation is requested, the time being the busy season. A full representation from every state in the United States is earnestly desired.

J. J. WRIGHT, *President*,
Wells Bldg.,
Milwaukee, Wis.

CHAS. A. MEEKER, *Sec'y*,
Fulton St.,
Newark, N. J.

### JUNE DENTAL MEETINGS.

CALIFORNIA STATE DENTAL ASSOCIATION. San Francisco. Four days: June 22d to 25th.

GEORGIA STATE DENTAL SOCIETY. Atlanta. Three days June 14th to 16th.

MAINE DENTAL SOCIETY. Rangeley. Three days: June 22d to 24th.

MARYLAND STATE DENTAL ASSOCIATION AND DISTRICT OF COLUMBIA DENTAL SOCIETY. Baltimore. Three days; June 9th to 11th.

MASSACHUSETTS DENTAL SOCIETY. Springfield. Three days: June 14th to 16th.

MICHIGAN STATE DENTAL SOCIETY. Detroit. Three days: June 13th to 15th.

NORTHERN OHIO DENTAL ASSOCIATION. Toledo. Three days: June 7th to 9th.

PENNSYLVANIA STATE DENTAL SOCIETY. Harrisburg. Three days: June 28th to 30th.

SOUTHERN CALIFORNIA DENTAL ASSOCIATION. Los Angeles. Three days June 16th to 18th.

EXAMINERS' MEETINGS.

ALABAMA BOARD OF EXAMINERS. Mobile. May 9th.

ARIZONA BOARD OF EXAMINERS. Tucson. April 18th to 21st.

CONNECTICUT DENTAL COMMISSIONERS. Hartford. June 16th to 18th.

ILLINOIS BOARD OF EXAMINERS. Chicago. June 13th.

MARYLAND BOARD OF EXAMINERS. Baltimore. May 19th and 20th.

MICHIGAN BOARD OF EXAMINERS. Ann Arbor. June 20th to 25th.

MINNESOTA BOARD OF EXAMINERS. Minneapolis. March 15th to 17th.

MISSISSIPPI BOARD OF EXAMINERS. Jackson. May 17th.

NEBRASKA BOARD OF EXAMINERS. Lincoln. June 27th.

NEW HAMPSHIRE BOARD OF REGISTRATION. Manchester. June 1st to 3d.

NEW JERSEY BOARD OF REGISTRATION. Trenton. July 5th to 7th.

NORTH CAROLINA BOARD OF EXAMINERS. Wrightsville. July 11th.

TEXAS BOARD OF EXAMINERS. Houston. June 13th.

WASHINGTON BOARD OF EXAMINERS. Seattle. May 26th.

WEST VIRGINIA BOARD OF EXAMINERS. Wheeling. June 8th to 10th.—*Cosmos.*

---

OHIO STATE DENTAL BOARD. The regular spring meeting of the Ohio State Dental Board will be held in Columbus on June 21–24, inclusive. All persons desiring to secure licenses at this time should secure blank forms of application from the secretary in time to return same by June 11th, together with the fee of twenty-five dollars.

For further information address

L. L. YONKER, *Sec'y*, Bowling Green, O.

---

IOWA BOARD OF DENTAL EXAMINERS. The Iowa State Board of Dental Examiners will hold a meeting for the examination of candidates for license to practice dentistry in Iowa, beginning June 6th, 1910, at 9 A. M., at Iowa City.

For blanks and other information, write

E. D. BROWER, *Sec'y*, Le Mars, Iowa.

---

CANADIAN DENTAL ASSOCIATION. The bi-annual meeting of the National Dental Association of Canada will be held in Toronto, May 31, June 1, 2, and 3, 1910. Papers and Clinics. We invite our American brethren, and offer them all the privileges of the convention. Delightful temperature in June. Toronto is three hours from Buffalo, four from Muskoka or Georgian Bay, one night from the famous Cobalt and six hours from the Thousand Islands of the St. Lawrence. Come and join us. Single fare rates, upon certificate plan, over all of Trunk Line Territory.

GEORGE W. GRIEVE, *Sec'y.*

The Post-Graduate School of Dentistry of Berlin. A Post-Graduate School for holders of German or foreign State certificates in dentistrv will be opened this April at 22 Bulowstrasse, Berlin.

The instruction will be in charge of the following dentists. Dr. Konrad Cohn; Professor Dr. Dieck; Professor Guttmann, Court Dentist; Professor Hahl; H. J. Mamlok; Professor Dr. Wilh. Sachs; Dr. Erich Schmidt; Professor Dr. Schroeder; Professor Dr. Williger, Willmer Court Dentist.

The new school will have 12 rooms at its disposal.

Its purpose is to give all its visitors an opportunity of doing advanced scientific and practical work, of acquainting themselves with the latest improvements and inventions in surgical, technical and operative dentistry, including Roentgen diagnosis in a specially equipped Roentgen laboratory and of familiarizing themselves with the application of these improvements by actual treatment of patients.

A dental infirmary is to be connected with the School so that there will be no lack of material for instruction.

Further information as to the Post-Graduate School will be given by Dr. Erich Schmidt, 133 Potsdamer Strasse, Berlin.

